# Scalable, Modular Three-Dimensional Silicon Microelectrode Assembly via Electroless Plating

**DOI:** 10.3390/mi9090436

**Published:** 2018-08-30

**Authors:** Jörg Scholvin, Anthony Zorzos, Justin Kinney, Jacob Bernstein, Caroline Moore-Kochlacs, Nancy Kopell, Clifton Fonstad, Edward S. Boyden

**Affiliations:** 1 Massachusetts Institute of Technology, Cambridge, MA 02139, USA; scholvin@MIT.EDU (J.S.); anthonyzorzos@gmail.com (A.Z.); jkinney@mit.edu (J.K.); jgbernstein@gmail.com (J.B.); caromk@gmail.com (C.M.-K.); fonstad@mit.edu (C.F.); 2 Department of Mathematics, Boston University, Boston, MA 02215, USA; nk@math.bu.edu

**Keywords:** electrode array, microelectrodes, neural recording, silicon probe, three-dimensional, electroless plating

## Abstract

We devised a scalable, modular strategy for microfabricated 3-D neural probe synthesis. We constructed a 3-D probe out of individual 2-D components (arrays of shanks bearing close-packed electrodes) using mechanical self-locking and self-aligning techniques, followed by electroless nickel plating to establish electrical contact between the individual parts. We detail the fabrication and assembly process and demonstrate different 3-D probe designs bearing thousands of electrode sites. We find typical self-alignment accuracy between shanks of <0.2° and demonstrate orthogonal electrical connections of 40 µm pitch, with thousands of connections formed electrochemically in parallel. The fabrication methods introduced allow the design of scalable, modular electrodes for high-density 3-D neural recording. The combination of scalable 3-D design and close-packed recording sites may support a variety of large-scale neural recording strategies for the mammalian brain.

## 1. Introduction

Silicon microfabricated neural probes [1,2,3,4,5,6,7,8,9,10] offer the capability of scalable neural recording in acute and chronic neuroscience experiments [8,9,10,11], since hundreds of, or more, electrode recording sites can be created on an implantable 1-D or 2-D shank using scalable microfabrication techniques. Recently we designed, implemented and used 2-D silicon microelectrode arrays bearing close-packed recording sites, designed with small enough spacing to enable spatial oversampling of extracellular action potentials—and thus, scalable, tetrode-style analysis to be performed on the data obtained [12]. Here we explore another key aspect of scalability, namely how to fabricate silicon microfabricated neural probes with electrode pads distributed in 3-D, not just 2-D, patterns. 1-D and 2-D microfabricated silicon probes primarily record in a small part of the brain and by design cover a one- or two-dimensional subset of the brain. Most silicon based probe technologies have the ability to record in 2-D, either in a vertical plane (“Michigan probes” [2]) with recording sites along each shank using in-plane microfabrication, or a horizontal plane (“Utah array” [13]) with recording sites at the shank tips, using bulk micromachining techniques.

### 1.1. Overview of 3-D Approaches

To record from an entire region of the brain, or across multiple regions simultaneously, the neural recording sites need to cover a 3-D volume. Therefore, a 3-D probe needs to consist of many shanks, each bearing multiple electrode recording sites that record at many points along its length. But, because microfabricated devices are currently inherently two-dimensional, combining them into a 3-D structure presents engineering challenges, such as the question of how to create mechanical and electrical connectivity between individual 2-D parts, in a scalable, modular way. Since the introduction of the first 3-D neural probes comprised out of individual 2-D parts [14], different technologies for assembling arrays have been explored: ultrasonic bonding [15,16,17], pressed contacts [18,19,20], solder reflow [21] (also used in early explorations for 3-D integrated circuits (ICs) [22]), conductive silver paste [23], post-packaging nickel or gold electrolytic plating [24,25], folding parts [26,27], self-assembly [28], electrostatic- [29] or magnetic-field [30] assisted assembly, die stacking with wirebonding [31] and different types of packaged stacking [10,32,33,34,35]. These solutions all share the principle of combining (or, in the case of [26,27,28,29,30], folding) individual 2-D probes in order to create 3-D arrays and are summarized in Table 1. The above studies primarily focus on pioneering new modalities of 3-D assembly. Our primary focus is to explore the scalability of 3-D assembly of modular microfabricated neural probes, aiming to develop robust, powerful methods for assembling probes bearing many thousands of electrode recording sites and beyond. We approach this by introducing electroless plating as a way of forming, in a simple single step, all of the electrical connections at the same time—thereby enabling a new efficient and scalable fabrication method.

Our methods can subsequently be combined with heterogeneous integration of amplifier circuits [36] to reduce the total number of actual wires leaving the device, for example, through wirebonds to a circuit board, because the external package size determines which in vivo recording scenarios the probe can be used in and can additionally govern the scale that is meaningfully achievable with a given probe design.

### 1.2. Creating a Scalable 3-D Probe Design

**Figure 1:**
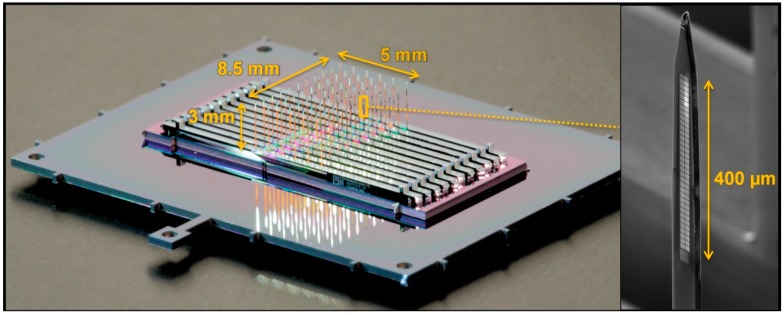
Photograph of a high-density 3-D probe, consisting of a 6 × 11 grid of shanks. Each shank contains a set of 2 × 34 close packed recording sites (as seen in the scanning electron microscope (SEM) inset), for a total number of 4488 sites across a volume of 5 × 8.5 × 0.4 mm.

Virtual reality awake head-fixed setups [39], for example, for mouse behavior, have become widespread in neuroscience because they enable neural recording and imaging during animal behavior experiments, without the weight and size constraints of freely moving animal behavior experiments [40]. A design example of a 3-D scalable probe appropriate for such an experiment is shown in Figure 1. The need for scalability is particularly important for 3-D probe arrays, because to tile a 3-D volume, one needs a far greater number of recording sites than required to tile a 2-D section.

We describe new principles for scalable, modular mechanical and electrical assembly of 3-D structures, using self-locking mechanical components that allow easy by-hand assembly and we introduce the use of electroless nickel (EN) plating to form orthogonal electrical connections between individual parts in a scalable way. The electrical connections are formed without the need for electrical access to the sites, relieving potential constraints on future monolithic or heterogeneously integrated neural amplifier circuits (as outlined in [36]). Our method therefore supports equally well passive and active probes (i.e., probes without and with integrated amplifiers or other circuitry, respectively) and is carried out prior to probe packaging, relieving constraints on the final packaging steps.

We utilize our close-packed 2-D probe technology of [12] as the unit building block for our 3-D arrays. With probe designs scaling possibly into the thousands of recording sites and beyond [41], the close-packed recording sites can be of benefit in automating the large-scale data analysis that will be necessary when recording from a large number of sites across many brain regions.

### 1.3. Scope of the Design

We focus this paper only on the design and fabrication challenges of probe arrays but not on the downstream packaging and in vivo testing—which will partly depend on the final application; such testing may then require refinement or alteration of the design depending on how well the probe performance matches the goal. For example, awake headfixed extracellular recordings in rodents can utilize probe arrays attached to printed circuit boards with conventional methods such as wirebonding or flip-chip assembly. In contrast, chronic applications may require flexible cables to be used (e.g., as described in [20,42,43,44,45]), although this may dictate a lower channel count due to packaging restrictions. Our goal is to demonstrate methods of creating highly scalable Si based electrode arrays and we accordingly uncouple their design from the packaging choice. But for highly-scaled 3-D probes aimed at in vivo headfixed recordings, we can draw on existing solutions used in the semiconductor probe card industry, where systems face even more complex packaging constraints and designs connect and route over 10,000 high-speed wires out from a small space to sophisticated test equipment [46]. The semiconductor industry roadmap also sets out to increase the maximum number of pins to around 50,000 by 2028 [47], with each connection supporting significantly higher bandwidth than a passive probe or active neural amplifier requires. Probe-card packaging technologies can inform us about the current technological limits relevant for awake headfixed experiments, where the size and weight of the setup is not a determining factor.

## 2. Materials and Methods

### 2.1. Fabrication and Assembly Overview

**Figure 2:**
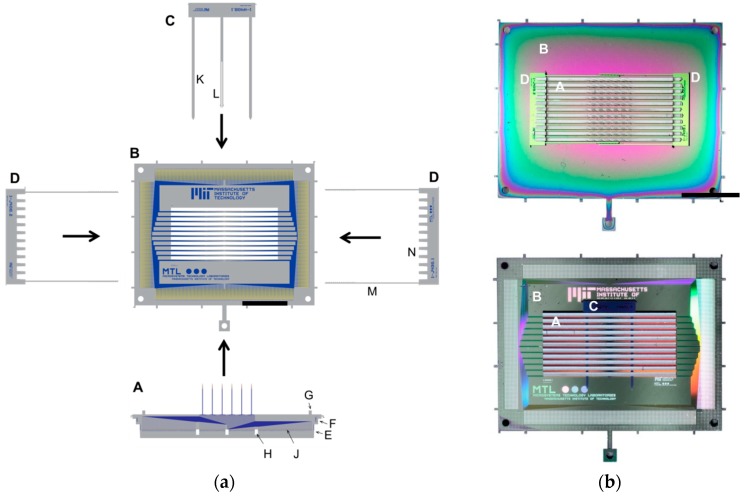
(**a**) Principle of the mechanical assembly for the probe shown in Figure 1. The 2-D inserts (A) are slid into the openings in the holder plate (B). A slight taper (E) on the 2-D insert facilitates the hand-assembly and a small bump (G) protects the shanks when pushing the 2-D inserts down with tweezers. On the bottom, a self-locking hook (C) with two guide beams (K) and locking beams (L) is then pushed through the openings in the 2-D inserts (H). A pair of self-locking alignment structures (D) is inserted on the top. Its tapered combs (N) push the 2-D inserts (A) into alignment and lock themselves into place: vertically confined by an indent (F) and horizontally by interlocking beams (M). The recording sites on the 2-D insert are wired down to the contact pads (J) and are described in the electrical assembly section. The fabrication steps for the individual parts are shown in Figure 3 and Figure 4. (**b**) The top and bottom photographs show an assembled device from above and below, respectively, with the individual components labeled. Scale bars are 10 mm. A typical step-by-step assembly sequence is: (step 1) lay out the individual components for assembly, (step 2) grip holder plate (B) with reverse tension tweezers, (step 3) sequentially pick up the inserts (A) with fine tweezers and insert through the slots in (B), (step 4) inspect and tap down with tweezers onto the inserts to make sure they are fully inserted into the holder plate, (step 5) pick up the self-locking hook (C) with fine tweezers and insert the guide beams (K) through the openings of the inserts (H). It can help to lift the reverse tension tweezers with the probe to better see, or place a mirror below the probe for visual guidance. (step 6) once the guide beams are inserted, use either tweezers or your finger to gently push the hook through completely. As the guide beams pass through each insert, a small resistance can be felt when pushing, due to the hooks (L) going through the openings (H) of each insert. (step 7) using tweezers, pick up the two alignment structures (D) and place on the top side of the probe body (B), roughly aligning them. (step 8) using two sets of tweezers, one in each hand, push the two structures (D) closer until they slide into position. The tweezers should be open in this step, allowing both pushing and rotating of the two parts (D) as they approach and lock. Avoid pushing both parts forward at the same time but alternate between them. Use your dominant hand for the last fine push that locks the beams of (D) together and aligns the probe.

The process for fabricating the silicon parts for the 3-D probes described in this paper is nearly identical to that for the 2-D probes previously reported [12]. We will refer to that work for detailed fabrication methods, while noting the differences here. The key innovation reported here involves the mechanical and electrical assembly of individual 2-D probes into a 3-D structure and the necessary layout changes required for accomplishing this. The overall principle of the mechanical assembly is shown in Figure 2.

We build a 3-D array from four types of components: individual 2-D probe inserts (point A in Figure 2a,b) which are placed into a slotted holder plate (point B in Figure 2a,b), similar in principle to [14,48]. The 2-D inserts as well as the holder plate contain electrical wiring and exposed pads for electroplating, packaging and neural recording. The electroplating pads (point J in Figure 2a) are later connected with electroless nickel plating. We identify the two sides of the holder plate according to how the array is shown in Figure 1, with the probe shanks pointing up. Thus, the top side of the holder plate is on the same side as the probe shanks, while the bottom side of the holder plate contains the pads and wiring. The choice for placing the pads on the bottom side of the holder plate is not critical but it helps to increase the space available to redistribute wires from the shank to the contact pads (point J in Figure 2a) within each 2-D insert. We can also imagine a holder plate with metal pads on both sides, in an effort to double the wiring density (or, for active 2-D probes, to assist in spatially isolating different signal types).

The 2-D inserts are placed into the opening slots from the top side. In contrast to our previous work on 3-D waveguide arrays [48], we introduce a self-locking hook (point C in Figure 2a,b) that locks the 2-D inserts into place. This hook is inserted on the bottom side of the holder plate, through a set of openings etched into the 2-D inserts (point H in Figure 2a). The hook is a simple deep reactive ion etched (DRIE) silicon structure. Finally, on the top side of the holder plate, a pair of self-locking tapered comb structures (point D in Figure 2a,b) is inserted to help with self-aligning the 2-D inserts to all point in the same direction.

**Figure 3:**
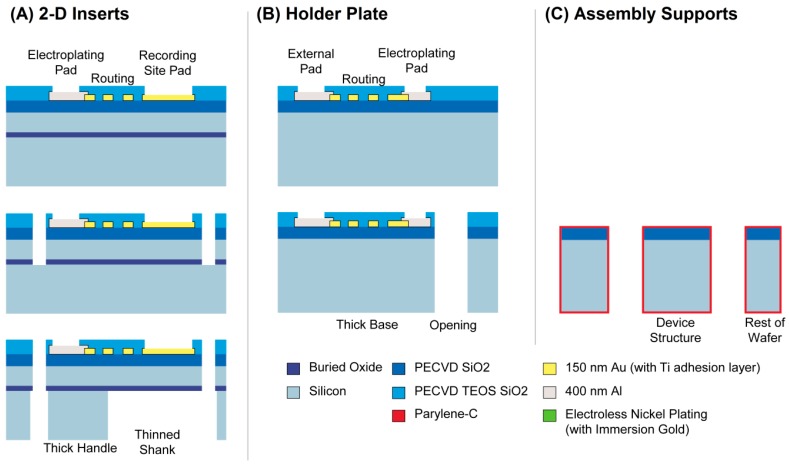
The three different design components, with photographs of the finished 150 mm diameter wafers (top) and process cross-sections (bottom). The cross sections are not to scale. Parts (**A**), the 2-D inserts, are fabricated identically to our 2-D probe components reported in [12] with the exception of using 400 nm Al as the optical lithography metal (instead of 250 nm Au). Part (**B**) is the holder plate and identical to A except that the DRIE etch consists of a single through-etch from the front-side, instead of a front- followed by a back-side etch in A. Finally, part (**C**) is a single silicon deep reactive ion etch (DRIE) step to create the self-locking hooks and the alignment comb structures.

**Figure 4:**
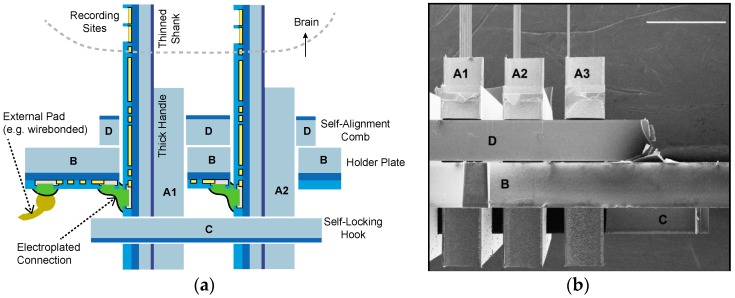
(**a**) Cross-section schematic of the assembled probe, with the different parts from Figure 3 labeled (drawing not to scale). (**b**) SEM image showing a close-up side-view of the probe in Figure 1, with the corresponding parts labelled. The scale bar is 1 mm. After completed assembly, the probe can subsequently be encapsulated in epoxy (not shown here), protecting the electroplated and external connections, while leaving the thinned shanks free from encapsulation (similar to how we epoxy-encapsulate our 2-D probes in [12]).

The different components and their respective cross sections are shown in Figure 3, along with a comparison of the process steps in Table 2. Once the parts are fabricated and mechanically assembled into a 3-D structure, electroless nickel deposition forms the electrical connections and prepares the probe for wirebonding or other packaging steps. To wirebond these probes, a dedicated wirebond chuck will need to be prepared, so that after attaching the probe to a printed circuit board (PCB), the shanks are protected. We used a similar approach when carrying out the electrical measurements on an assembled probe (using an aluminum block with a recessed area for the shanks). The cross section and side-view of an assembled probe is shown in Figure 4. The next two sections explain the design and process choices made for the mechanical and electrical assembly.

### 2.2. Mechanical Assembly Procedures

#### 2.2.1. Inserts and Holder Plate

The mechanical assembly is designed to be simple and done by hand using mechanical tweezers, without a need for robotic assembly. The holder plate (point B in Figure 2a,b) can be fixed in space using forceps or by using a customized holder. The individual 2-D inserts (point A in Figure 2a,b) are placed into the holder plate slots, which is an easy task as long as these openings are wider by about 5 to 10 µm. The inserts have tapered sides (point E in Figure 2a) to allow initial misalignment when inserting them into the openings. Once inserted, we tap down on the inserts with tweezers and ensure they are placed all the way into the holder plate.

#### 2.2.2. Self-Locking Hook

Once all of the 2-D inserts are in place, a self-locking hook is inserted on the underside of the holder plate, locking all of the 2-D inserts into position (point C in Figure 2a,b). We use a design with at least three beams. The pair of outer beams is longer and solid and they function as guide beams (point K in Figure 2a). The inner beam(s) each contain a pair of locking beams (point L in Figure 2a), which will self-lock once inserted through the 2-D probe openings (point H in Figure 2a). The purpose of the guide beams is to enter first and align the insertion. Without the guide beams, it is very difficult to insert the hook by hand, because any off-angle insertion will easily break the fragile center hook pair. Any initial misalignment is self-corrected by the guide beams and they are strong enough to not break during this process. Once the guide beams are inserted, the self-locking hook can be pushed through, either using tweezers or the tip of a finger (this has the benefit of feeling the changes in mechanical resistance when the locking beams pass each insert—helpful at least initially when practicing the assembly).

The geometry of the locking beams was initially chosen using a simple cantilever beam formula (to get the necessary displacement of the tips yet stay well below the stress limits of silicon) and then experimentally optimized using a range of different designs. In our current designs, the locking beam’s length is always identical—with an aspect ratio of L/W = 4500 µm/95 µm = 47. During insertion, the hook tip is displaced by as much as 50 µm, resulting in a maximum simulated stress of 0.07 GPa (calculated using finite element methods), well below the silicon fracture strength of around 1.5 to 2.0 GPa [49].

#### 2.2.3. Self-Alignment Combs

The final step in the mechanical assembly is to insert a pair of self-aligning and self-locking tapered combs on the top side of the holder plate (point D in Figure 2a,b). We introduced these structures in [48] but show important improvements in their design here.

Because the openings in the holder plate must be slightly larger than the 2-D inserts placed through them, it is possible for each insert to point in a slightly different direction. The purpose of the tapered combs is to prevent misaligned shanks because they can result in excessive tissue damage. The opening will be larger than the insert for three reasons: First, there can be variations in the 2-D insert’s thickness caused by wafer thickness variations. But these are small and can be adjusted for in the design, thus posing no major concern. Second, variations can be by design. The dimensions of the opening in the holder plate are lithographically defined and thus set precisely. However, to facilitate insertion, some tolerance is necessary (e.g., on the order of 10 µm for hand-assembly). Third, variations can be due to non-vertical sidewalls of the etched openings, which is harder to control. The sidewall angles of the DRIE depend on the tool and recipe optimization and can add significant uncertainty. When etching through 525 µm thick wafers, the bottom of the trench is wider than the top; we observed around 30 µm of widening (or a 3.3° tilt from the vertical). The precise value can vary with tool condition and etch recipe parameters. While recipe optimization could reduce the trench widening, an effective solution is to etch half-way through the wafer from each side with the trenches meeting up in the middle (e.g., as done in [15]). This adds another lithography step and some front-to-back alignment uncertainty (up to a few µm) can remain. Even with robotic assembly and near-perfect etch precision, small non-vertical DRIE sidewalls or process misalignments can still allow probes to rotate. Consequently, the alignment structures remain beneficial even when assembly and process conditions are improving and their presence allows us to avoid challenging optimization and monitoring of processing tolerances and instead build in a 5 to 10 µm gap that greatly facilitates assembly.

#### 2.2.4. Improvements to the Self-Alignment Combs

**Figure 5:**
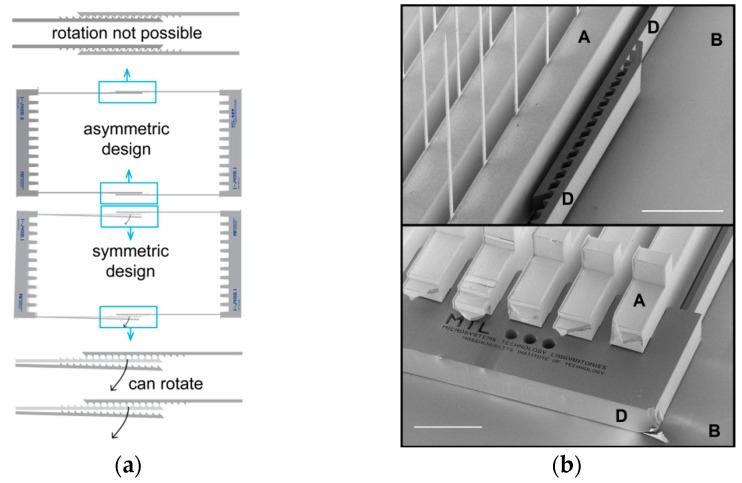
(**a**) Different locking designs for the self-alignment combs. For narrow distances, the symmetric design is acceptable but as the two combs are spaced further apart, rotations can cause problems and an asymmetric design instead is preferred. (**b**) The SEM on the right shows detailed views of the tapered comb (D) locked between the 2-D inserts (A) and holder plate (B) in the bottom image and its beams (D) locked onto itself in the top image. The scale bar is 1 mm.

Our initial use of the self-alignment combs in [48] was a single design of alignment beams, where two identical structures self-interlocked. Rather than having many hook pairs between each insert (as done in [48]), we found two interlocking hooks located at the ends of the structure were sufficient to provide mechanical stability (point M in Figure 2a and Figure 5). Placing the interlocking hooks only at the ends allows us to reduce the pitch between the 2-D inserts.

The symmetric interlocking hook design is suitable for smaller inserts (e.g., inserts of 5 mm size). However, for larger inserts such as the ones we are presenting here (the base of the insert in Figure 1 is 1.9 cm wide), a problem arises: when sliding the structures into place, the symmetry of the interlocking structures means that small rotations in one direction can result in a lack of self-locking (bottom of Figure 5a). We adjusted the design to use asymmetric structures: one with the locking beams on the outside and another with the beams on the inside (top of Figure 5a). This removes the freedom to rotate and ensures that the structures will stay interlocked in place since small rotations will be counteracted in either direction.

A further improvement made relates to the contact between the self-alignment combs and the 2-D insert. The comb structure has a slightly tapered shape, so that the comb gradually presses against the 2-D inserts. If the surfaces touching are both silicon, they cannot be pressed well into each other and their points of contact will be minimal. We decided to coat the combs with a 1 µm layer of Parylene-C, to provide a thin, soft coating on the alignment combs. When pressed against the silicon 2-D inserts, the soft Parylene-C provides a press-fit type mechanism. Pushing in the alignment combs becomes easier and adds stability as the point of contact between the combs and the inserts is now larger. We chose Parylene-C because it is easy to deposit uniformly on finished wafers with DRIE through-etched patterns. We use Parylene-C only to provide a press-fit coating for the assembly and do not deposit it on the shanks, or any other neural recording related parts of the probe.

#### 2.2.5. Alternative Assembly Methods

The assembly described above is done by hand but a robotic or micromanipulator based assembly could be developed in the future and would allow reduction of tolerances, useful for more aggressively scaled electrical connections. Our initial 2-D insert design had additional features, which we eventually omitted from the final design. However, we describe these initial design features briefly here, because they may become relevant in the future if robotic assembly is used.

**Figure 6:**
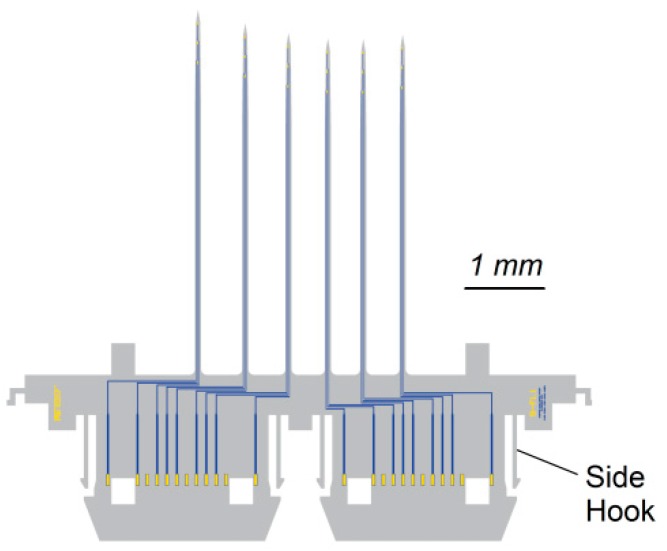
Design drawing of a 2-D insert with side-hooks.

In our initial design, the 2-D probe inserts were not locked into place until the bottom hook was inserted. If, however, the inserts needed to be held in place temporarily, a small set of hooks could be included, as shown in Figure 6. These smaller side-hooks snap into place when the 2-D insert is inserted through the holder plate. The single large opening shown in Figure 2 can also be interrupted with a number of bridges, to utilize more than two side-hooks. These bridges can also help give mechanical strength to the holder plate if the pitch between inserts is very small, although we did not notice that to be a problem. However, we discovered that the main problem with the side-hooks is their fragility (being very thin yet stiff beams). During manual assembly, the natural shaking of the hand resulted in an estimated two-thirds of the side-hooks breaking. By itself, this may not be problematic but the side-hook length is much larger than the wafer thickness and broken side-hooks therefore create a significant challenge: with the hook broken, the 2-D insert now has substantial space to move around, easily creating a horizontal misalignment between the electrical pads on the holder plate and those on the 2-D insert. This can make the electrical assembly impossible. Thus, we decided not to use the bridges or side-hook concepts and instead went with a simpler, tapered insert design. A robotic assembly method may however find the side-hooks beneficial, because robotic precision may avoid breaking them.

### 2.3. Electrical Assembly Procedures

**Figure 7:**
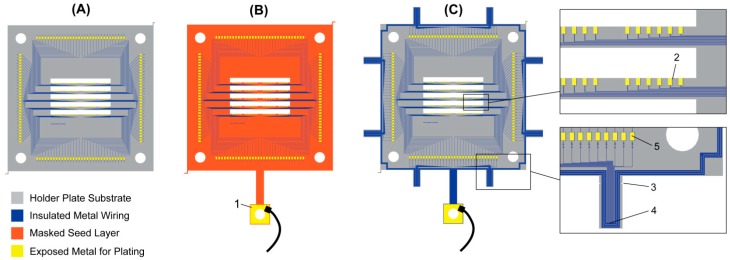
Layout illustrations for the different electroplating methods: (**A**) packaged and electroless plating, (**B**) seed and masked plating and (**C**) design with short-circuit beams. All cases aim to electroplate the small pads near the opening in the holder plate, shown as (2) but differ in how plating current is supplied to these pads. The larger external contact pads (5) can be plated as well, facilitating final packaging. The design in (A) has the lowest complexity. In (B), a seed is masked with exposed pads only at the desired plating sites. Plating current is then supplied at the contact (1), for example, by attaching a temporary clip. For short-circuit beam plating (C), no seed layer is used but instead all sites are routed and connected to a wiring frame (4), which consists of several wiring rings, resulting in a tree-like structure to balance the voltage drop for each pad. After plating, the shorts are disconnected by breaking off the external short circuit beams at (3).

To electrically connect the 2-D probe inserts with the holder plate, a connection across a gap and between pads on two orthogonal surfaces (the holder plate and the 2-D insert) must be made. Our goal was to create a scalable approach that could easily form thousands of connections. The mechanical constraints (because the points of connection are in a “canyon” which does not allow easy mechanical access) rule out ultrasonic bonding as a practical method. We also decided against solder based methods, because we thought that connections at a pitch at tens of microns would be extremely challenging and because sample preparation with solder would require additional process steps prior to DRIE. Instead, we focused on different ways of electroplating to form connections (e.g., similar to [24]). The layout for each method is shown in Figure 7 and we compare their merits in Table 3.

#### 2.3.1. Post-Package Electrolytic Plating

In packaged plating (e.g., [24,25]), the probe is first assembled and packaged (e.g., to a PCB), so that individual sites on the holder plate can be electrically accessed through the package connector. This allows flexibility in the plating: each pad can be individually plated, or pads can be plated in parallel. Individual plating can allow end-point detection of the plating but this approach does not scale well with the number of pads. Because the probe must first be completely packaged, any electroplating yield problems will not be identified until after packaging, increasing the time and cost caused by non-yielding devices. The package also needs to be compatible with the electroplating chemicals.

#### 2.3.2. Electrolytic Plating with Seed Layer and Mask

A common approach for electroplating in microfabrication is to use a masked seed layer to plate from, shown in Figure 7B. The seed blankets the entire device (in our case the holder plate) and is selectively covered by an insulating mask with only the desired plating sites exposed. Photoresist is often used as a convenient mask material. By plating with a seed and mask, a single point of contact to the seed can supply the plating current for all sites in parallel, independent of what devices or wiring is implemented in the actual silicon below the seed. After plating, the mask and seed are chemically removed. In our process, the mask and seed must be fabricated prior to the DRIE etch. Therefore, it is not possible to use photoresist as a plating mask. This restriction complicates the choice of mask material. We initially implemented this approach with electrolytic Cu plating using a thin evaporated Cu seed (e.g., 100 nm), masked by a thin film (e.g., 150 nm) of plasma-enhanced chemical vapor deposition (PECVD) SiN_x_ or SiO_2_. The Cu seed required either Ti or Cr to be used as an adhesion layer (e.g., 10 to 20 nm). However, our choice of Cu was not ideal, because of metal adhesion and removal issues (caused by the presence of difficult to remove Cu/Cr or Cu/Ti intermetallics). The mask layer removal in diluted HF was also not ideal, because it attacked the probe insulating dielectric films. Thus, switching from Cu to Ni or Au as the seed and plating metal could help to reduce some of these problems. But the requirement for chemical etching to remove the mask would remain.

#### 2.3.3. Seedless Plating with Temporary Short Circuits

**Figure 8:**
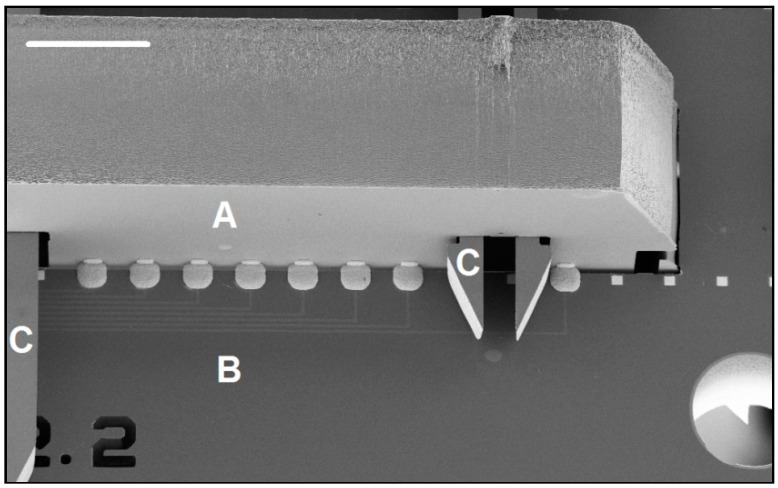
SEM image of an earlier electrolytic plating design, showing 2-D insert (A), holder plate (B) and self-locking hook (C). The scale bar is 400 µm.

We also investigated an intermediate step between packaged plating and plating with a seed and mask (Figure 7C and Figure 8). In this approach, we modify the wiring layout on the holder plate and short-circuit all of the pads together, so that they can be plated in parallel. This method removes the need for a seed or mask layer and also the need to first package the device. The short-circuits must be temporary and we route the wiring to the outside of the holder plate, where they are short-circuited together and connected to a single plating access site. This approach requires balancing of the line resistance and we implemented a tree-like structure along the short circuit ring’s perimeter. If a simple short circuit ring is used, distant plating sites will fail to plate due to significant potential drops along the way. After plating, short-circuits are removed by physically cleaving the beams with side-cutting pliers. The disadvantage we found with this method is a lack of scalability and added design complexity. The space on the short circuit beams is fixed, requiring either a larger number of wider beams, or finer metal traces as we scale up the pad count. We also found that breaking off the short circuit beams poorly can sometimes result in broken metal wires short-circuiting because of the ductility of the metal. An alternative may thus be to use laser-cutting rather than cleaving.

#### 2.3.4. Electroless Plating

The previous methods all relied on electrolytic plating, where plating current is supplied externally. In contrast, electroless (or autocatalytic) plating is able to deposit metal without the need for an external current supply. This method is ideal for our designs, because it minimized the process complexity and avoids the handling complexities to make a temporary single electrical contact on the assembled 3-D probe. The design of the holder plate is shown in Figure 7A. We decided to use electroless nickel (EN) plating, because it provides a well-established plating protocol compatible with plating on Al pads. We use a standard phosphorous nickel solution (Fidelity 9012, OMGroup Inc., Piscataway, NJ, USA). The process starts with a pre-treatment for Al substrates: 5 min in OMG 3152 soak cleaner, 15 seconds in OMG 3133 acid etch, 30 s de-smut in 50% v/v nitric acid and 25% v/v sulfuric acid, 60 s in OMG 3116M zincate, 30 seconds de-smut in 50% v/v nitric acid, 60 seconds OMG 3116M zincate—with deionized (DI) water rinsing between steps. All solutions are mixed and operated according to the manufacturer’s specifications. The pre-treatment prepares the Al pads with a seed layer of zinc and is followed by EN plating. The plating time depends on the gap that needs to be bridged but will typically be between 30 and 60 min.

**Figure 9:**
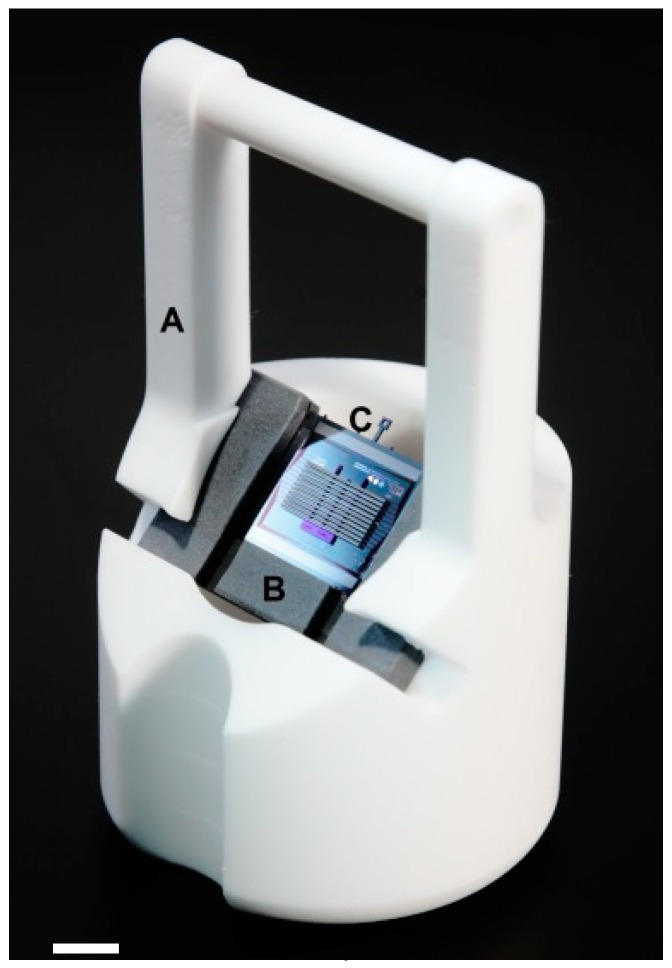
Teflon-based holder (A) for electroless plating. The darker insert (B) is specific to the probe (C) and holds the assembled probe at a 45-degree angle, to facilitate the removal of evolving gas from the plating sites to avoid gas getting trapped under a horizontal surface. The insert (B) can also be used to hold the probe during mechanical assembly steps (see section). The scale bar is 1 cm.

To hold the probe during plating, we use a Teflon carrier, custom-made by the Massachusetts Institute of Technology (MIT) Central Machine Shop (Figure 9). The carrier allows easy handling while protecting the probe and accommodates different inserts, one for each holder-plate design. In the carrier, the probe is held at a 45° angle to facilitate H_2_ gas evolution during plating and to avoid getting evolving gas trapped on or under a horizontal surface.

While the main purpose of the plating is to form the connections between the holder plate and the 2-D probe inserts, the external wiring pads also are plated (see Figure 7). Once the EN plating is complete, we follow with an immersion-gold step (Bright Electroless Gold, Transene, Danvers, MA, USA) to protect the Ni from corrosion and to make the external pads packaging compatible (e.g., for wirebonding).

Electroless nickel does not catalyze on the materials present on the probe shanks (Au recording sites, SiO_2_ insulator, Si shank). It is nonetheless a good idea to protect the probe shanks during plating with photoresist and removing the resist with acetone when plating is complete (as also suggested by [24]). Otherwise, stray Ni deposits may occasionally form especially on rough surfaces—for example, the DRIE sidewall or indented Au pad may trap contaminants in the plating bath that can then act as plating seeds.

#### 2.3.5. Comparison

**Figure 10:**
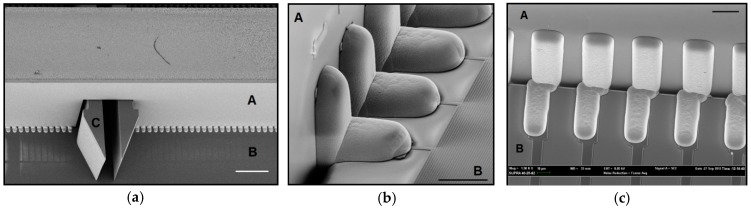
(**a**) SEM image of the 40 µm pitch connections of a 4480 recording site probe (design 3b in Table 4), with the 2-D insert (A), the holder plate (B) and the self-locking hook (C). (**b**) Electrical connections and shown with more detail. (**c**) An earlier test-design, also using a 40 µm connection pitch, with a narrow gap indicates the potential for a much narrower connection pitch. Scale bars are 200 µm (a) and 20 µm (b,c) respectively.

The above electroplating methods are summarized in Table 3. Based on our experience with the different methods, we find that electroless plating can enable scalable probe fabrication, by minimizing microfabrication process complexity and because plating can occur before packaging all in a single step regardless of the number of connections to be made. EN plating is also compatible with integration or attachment of complementary metal–oxide–semiconductor (CMOS) integrated circuits (e.g., similar to [7,17]) to the holder plate, because no electrical access to the plating sites is needed. However, the plating process is sensitive to substrate cleanliness. Organic contamination (e.g., from photoresist residues or the DRIE handle-mounting) must be properly cleaned, otherwise some pads may not plate. Regardless of the plating method used, the finest connection pitch that can be achieved depends on the gap that has to be bridged. The minimum pitch depends on the width of the pad and the spacing. Electrolytic plating needs to grow metal for a distance of W_gap_ (see Figure 8) and the minimum spacing to avoid short-circuiting neighboring pads is 2W_gap_. In contrast, electroless plating bridges the gap from both sides (Figure 10) and only W_gap_/2 is plated per side, giving a minimum spacing of W_gap_. These dependencies are summarized in Table 3.

We currently first assemble the 3-D probe before plating any of the parts. If a larger number of probes are required, it may become more time-efficient to initiate plating on the entire wafer—cleaning, zincate pre-treatments and a thin (e.g., 1 µm) initial EN plating—before breaking out the structures and assembling them into individual 3-D probes. After assembling the pre-plated structures, the probe can then be immediately placed into EN plating to form the final connections, simplifying the post-assembly processing.

We found that the protrusions at the end of the holder plate in Figure 7C make handling the device much easier and we introduced smaller protrusion on the edges of all our holder-plate designs. Similarly, the long bridge for seed/mask plating (Figure 7B) provides an easy way to pick up the device with tweezers. Our final designs use EN plating and while functionally equivalent to Figure 7A, have the physical outline of the holder plate in Figure 7C.

## 3. Results

### 3.1. Example Designs

We have built a number of different sample designs, summarized in Table 4. Each shank has close-packed recording sites (except for designs B160-F30 and B10-F10). The connection pitch ranges from a conservative 60 µm (B160-F160) to an aggressive 26 µm pitch (B1000-F1006, B1000-F1010). Testing was carried out on design 6, with was created with the goal of testing the minimum electroplating pitch and to demonstrate an aggressive number of very narrow shanks to test the DRIE etch capabilities. The following sections show the mechanical and electrical characteristics of these designs.

### 3.2. Mechanical Characterization

**Figure 11:**
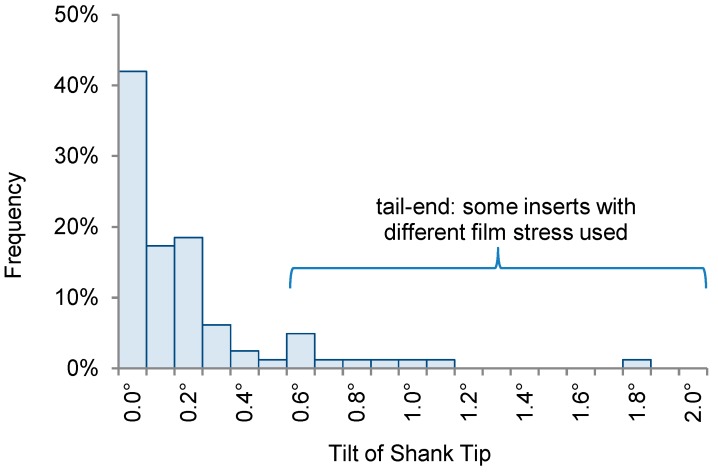
Measurements of the alignment accuracy based on six assembled 3-D probes. The strongly tilted shank tips were due to probe parts from wafers with different film stresses, resulting in an increased misalignment due to shank bending. For inserts with identical film stress, the shank tips are pointing within ±0.2° of their mean, equivalent to the tip of a 5 mm long shank being within 20 µm of the intended position.

To measure the alignment accuracy of the probe shanks, we mapped out the probe tip locations under an optical microscope with a digital stage. This allowed us to track the actual versus expected locations of the shank tips, with results shown in Figure 11. For these measurements, we placed a 3-D probe under the microscope and moved the stage in well-defined steps. At each step, we took microscope images focused on the shank tips and processed the results by measuring the actual versus expected pixel location of the tip, with a resulting measurement accuracy of around 3 µm. After mapping all of the probe’s inserts, we moved the stage back to the first position to verify that the probe had not shifted relative to the first image. Based on the characterization of six probes of three different designs, we find that the self-alignment method is effective with less than 0.2° misalignment. However, we noticed several probe inserts where the position was significantly different from its expected value (seen as the tail-end in Figure 11). Closer inspection of these probes revealed that they were using 2-D inserts from two wafers that had very different dielectric film stress (caused by a temporary tool problem that resulted in high stress instead of zero-stress in the final 1 µm tetraethoxysilane (TEOS) film deposited). While the inserts were well aligned, the probe shanks had different radii of curvature depending on the wafer they came from. Therefore, either all of the inserts should come from the same wafer or batch, or the shank bending should be characterized with an optical profilerometer before the devices are broken out of the wafer. Ideally, though, the dielectrics should be properly stress-balanced with minimal bending, thus avoiding the mismatch we encountered.

### 3.3. Electrical Characterization

Because the electrode recording sites are identical to that of our previously published 2-D designs [12], we here focus on the electrical and mechanical connectivity from 2-D to 3-D. Electrical measurements of line resistance and parasitic capacitance for the individual 2-D components, using a microprobe station and an impedance meter (HP4284A), are shown in Table 5. Measurements of the line resistances were done by connecting some of the ends of adjacent wires together on each component. 

We tested electrically assembled 3-D probes by measuring the resistance of wire pairs running from the holder plate and connected together on the 2-D insert. The electroplated contacts showed a resistance of 43 Ω. Wire pairs not connected were confirmed to be open circuits.

**Figure 12:**
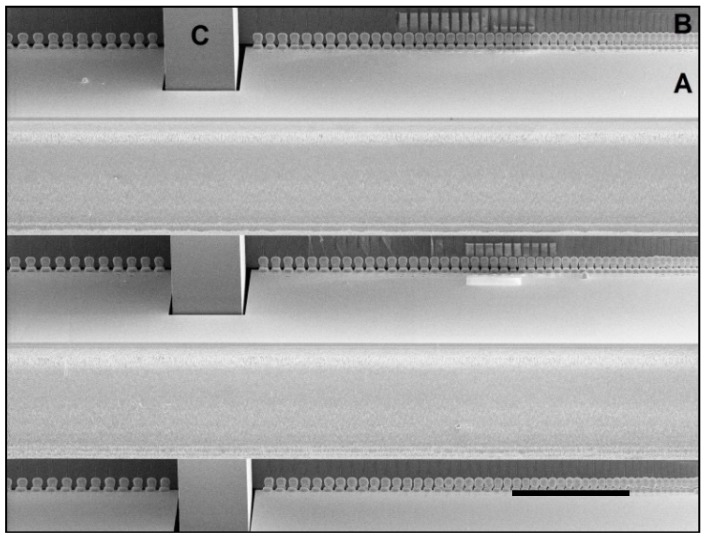
Example of a plating pitch test, showing several 2-D inserts (A) after electrical connections were formed to the holder plate (B). The self-locking hook (C) is visible. Each insert has a very fine pitch on the right and then relaxes the pitch towards the left. The finest pitch pads are visibly merged and overplated. Pads towards the left are clearly separated. The transition in this example occurred at around 35–40 µm but depends on the amount of plating necessary to bridge the gap (see Table 3). Scale bar is 500 µm.

To characterize the smallest electroplating pitch, we used a test design where each insert had a graduation of pitch values ranging from 16.5 µm to 113.7 µm (partially shown in Figure 12). We found that a pitch of 35 to 40 µm was easily achieved. The 26 µm pitch of the aggressive design turned out to be slightly too aggressive to allow successful contact formation with the 10 µm tolerance gap used. An SEM of the electrical connections of our standard design (design 3b in Table 4 and shown in Figure 1) with a 40 µm pitch is shown in Figure 9. We believe that by reducing the width of the pads and reducing the opening gap, a connection pitch down to 25 µm should be feasible (e.g., based on using an 8 µm wide pad and an 8 µm gap, which allows for a good overplating tolerance). Further increases in the number of connections may also be achieved by widening the base of the 2-D inserts (rather than reducing the pitch between connections).

## 4. Discussion

In this paper, we have focused on creating a scalable architecture for 3-D microelectrodes for neural recording. Scalability was demonstrated for both for the number of shanks and the number of recording sites per shank. Increases in the number of recording sites per shank are generally achieved through innovating the microfabrication process (the focus of our previous work [12]), while increases in the number of shanks requires a simultaneous innovation of scalable mechanical and electrical assembly methods, which has been the focus of this paper.

Increasing the number of shanks on an individual 2-D insert is relatively simple, because only adjustments in the layout need to be made. Increasing the number of 2-D inserts is possible as well, yet requires a tighter pitch between inserts. This can be achieved by using thinner 2-D inserts. The inserts can be entirely thinned back by the second DRIE etch step, similar to how the shanks themselves are thinned in our existing process—resulting in an identical thickness (here, 15 µm) of both the shanks and the 2-D insert’s base. However, this introduces conflicting requirements between the desire for thin shanks and a thicker base to simplify handling and avoid thin-film stress-induced bending of the base. But, these requirements can be uncoupled: by inserting a back-thinning step prior to the second DRIE, one can allow the base portion of the 2-D insert to be thinned back independently from the shanks and thus achieve an optimal thickness for both the shank and the base. For example, a base thickness of 50 to 100 µm may provide a suitable tradeoff between an array with tight pitch (e.g., 150 µm) and the mechanical stability to handle the inserts in assembly process (e.g., the waveguide arrays built in [48] used inserts of 50 µm thickness).

With automated layout generation (see [12]), many different probe geometries can be included on the same wafer (see Figure 3). This will enable us to readily create many designs that are targeted to different specific applications. Each 3-D probe can also consist of a set of unique 2-D inserts to span a more complex, or behavior-specific brain region (e.g., as demonstrated by [10]). The placement of recording sites can be individualized for each shank and the location and length of each shank can be tuned to perfectly suit a specific neuroscience goal.

The mechanical properties of Si shanks allow further scaling of the cross-sectional geometries [50], enabling a larger number of shanks for a constant tissue displacement of, for example, under 1%. Previous multielectrode designs have successfully used tissue displacement ratios of 2% for 100 Si shanks [13] and 2.8% for 30 microwires [51] in vivo and designs as low as 0.1% were suggested in [14]. Rapid insertion has been used for 100-shank designs with cone-shaped tips [52]. However, both the shape of the tip and the diameter of the shank have a strong impact on the optimal insertion speed and thus the chisel-tip geometries of the shanks presented in this paper may enable a slow and gradual insertion, similar to the design and analysis described in [53].

## 5. Conclusions

We have demonstrated fabrication and assembly technologies for 3-D probes and showed new methods to build scalable and close packed electrode recording sites, focusing on scalability of both the number of shanks as well as the number of recording sites per shank. Advanced packaging technologies in the semiconductor industry can enable these probes to be connected to and used for awake headfixed recordings in the future. The use of scaled 3-D probes in vivo will benefit from recent advances in surgery and experimental design (e.g., [39,54]) that will allow scaling up the number of recording sites without the constraints that chronic implant systems require.

The designs we introduced may help with explorations of the scalability of 3-D microelectrodes and the possibilities of wafer thinning and amplifier/multiplexer integration (either monolithic or heterogeneous) points towards a continued ability to scale in the future. A combination of these methods with our 3-D array assembly can significantly increase the number of recording sites beyond what we introduce here, we envision by two orders of magnitude or more, for the same number of external wires. Such scaling will bring along new engineering design challenges in packaging, thermal and power management and experimental design and data analysis. But the promise of extreme scalability is the capability to record a significant portion of the neurons in the mouse brain, obtaining and analyzing orders of magnitudes more information than currently possible.

## Figures and Tables

**Table 1:** 3-D Probe Fabrication Technologies.

Reference	Method ^a^	Design ^b^	Total Sites	Connection ^k^ Count Pitch	
Nordhausen 1996 [9]	Monolithic	10 × 10 × 1	100	n/a	n/a
Hoogerwerf 1991 [14]	Electrolytic	4 × 4 × 16	256	16 ^g^	-
Hoogerwerf 1994 [24]	Electrolytic	4 × 4 × 8	128	16 ^g^	-
Barz 2013 [25]	Electrolytic	4 × 4 × 4	64	64	70 µm
Herwik 2009 [18]	Pressed	4 × 4 × 5	80	80	70 µm
Kisban 2010 [20]	Pressed	2 × 4 × 5	80	80	35 µm ^d^
Aarts 2011 [19]	Pressed	4 × 4 × 5	80	80	70 µm ^e^
Bai 2000 [16]	Ultrasonic	4 × 4 × 4	32	32 ^g^	-
Yao 2007 [17]	Ultrasonic	4 × 8 × 32	1024	32 ^g^	-
Perlin 2008 [15]	Ultrasonic	4 × 4 × 4	64	64	40 µm
Malhi 1987 ^c^ [22]	Solder	9 × 1 × 22	198	198	-
Cheng 2014 [21]	Solder	5 × 4 × 5	100	100	150 µm
Lee 2009 [23]	Silver Paste	4 × 4 × 1	16	16	800 µm ^e^
Takeuchi 2004 [30]	Folding	2 × 3 × 3	18	n/a	n/a
Wang 2010 [28]	Folding	2 × 2 × 4	32	n/a	n/a
John 2011 [26]	Folding	3 × 3 × 2	18	n/a	n/a
Chen 2011 [29]	Folding	2 × 2 × 2	8	n/a	n/a
Merriam 2011 ^a^ [27]	Folding	4 × 4 × 4	64	n/a	n/a
Chiou 2010 [31]	Die Stacking	4 × 4 × 4	64	n/a	n/a
Rios 2016 [37]	Die Stacking	4 × 4 × 64	1024	256	200 µm
Du 2009 [33]	Package	4 × 2 × 8 ^h^	64	n/a	n/a
Langhals 2009 [35]	Package	4 × 4 × 4	64	n/a	n/a
Merriam 2011 ^b^ [32]	Package	5 × 4 × 8	160	n/a	n/a
Barz 2014 [34]	Package	2 × 2 × 8	32	n/a	n/a
Barz 2017 [34]	Package	2 × 2 × 8	32	n/a	n/a
Shobe 2015 [10]	Package	4 × 4 × 64 ^f^	1024	n/a	n/a
Michon 2016 [38]	Micro-Drive	16 × 2 × 8	256	n/a	n/a

^a^ Abbreviated methods, we define as “Package” assembly a method that uses non-microfabricated parts to combine 2-D probes. ^b^ Inserts/probe × Shanks/insert × Sites/shank. ^c^ Not used in a neural probe but relevant 3-D IC exploration. ^d^ Design conditions had a very strong impact on connection yield. ^e^ Not specified in the paper but inferred from images and drawings or previous work. ^f^ Design varies slightly from a uniform 4 × 4 × 64 configuration to accommodate brain region under study. ^g^ Active probe that uses multiplexing to reduce the connection count. ^h^ Double sided shanks. ^k^ The need for fine-pitched connection will vary, depending on the total number of recording sites, the target animal model and recording volume (which sets the space in which the connections must be made).

**Table 2:** Overview of Processing Steps. Summary of the process steps to fabricate the components of Figure 3. The process is adopted from and uses the same tools as our 2-D probes in [12].

Step	2-D Inserts (A)	Holder Plate (B)	Mechanical Supports (C,D)
Starting material	150 mm SOI wafer, thicknesses: 15 µm device layer, 0.8 µm buried oxide, 510 µm handle	150 mm wafer, 525 µm thick, double-sided polished	150 mm wafer, 525 µm thick, double-sided polished
Clean wafers and insulation	Piranha clean 1 µm of PECVD SiO_2_	Piranha clean 1 µm of PECVD SiO_2_	Omitted
Electron beam lithography metallization (liftoff)	10 nm Ti/150 nm Au/5 nm Ti, mask is 400 nm of PMMA 495A8	10 nm Ti/150 nm Au/5 nm Ti mask is 400 nm of PMMA 495A8	Omitted
Optical lithography metallization (liftoff)	50 nm Ti/400 nm Al, mask is 1.5 µm of AZ5214E	50 nm Ti/400 nm Al mask is 1.5 µm of AZ5214E	Omitted
Upper insulation	1 µm of PECVD TEOS	1 µm of PECVD TEOS	Omitted
Electron beam lithography small recording site etch	CF_4_/CHF_3_ based SiO_2_ etch, mask is 800 nm of PMMA 495A11	CF_4_/CHF_3_ based SiO_2_ etch, mask is 800 nm of PMMA 495A11	Omitted
Optical lithography large pad etch	CF_4_/CHF_3_ based SiO_2_ etch, mask is 1 µm of SPR-700	CF_4_/CHF_3_ based SiO_2_ etch, mask is 1 µm of SPR-700	Omitted
Frontside DRIE etch ^a^	CF_4_/CHF_3_ based etch of frontside SiO_2_, then 15 µm etch of Si device layer to buried oxide. Mask is 8 µm of AZ4620.	CF_4_/CHF_3_ based etch of frontside SiO_2_, then 15 µm etch of Si device layer to buried oxide. Mask is 8 µm of AZ4620.	Etch 525 µm through wafer, mask is 8 µm of AZ4620
SOI wafer buried oxide etch	CF_4_/CHF_3_ based etch of 0.8 µm of buried oxide.	Omitted	Omitted
Backside DRIE etch	Etch 510 µm through wafer from the backside, mask is 8 µm of AZ4620	Omitted	Omitted
Clean wafers	Barrel ash in oxygen plasma	Barrel ash in oxygen plasma	Barrel ash in oxygen plasma
Insulation on full wafer	Omitted	Omitted	0.1 µm ^c^ of PECVD SiO_2_ 1 µm of Parylene-C
Remove parts	Break out devices	Break out devices	Break out devices
Insulation on individual parts	(optional) Place dies facing down onto a Si wafer and deposit 100 nm PECVD Si_3_N_4_ to insulate backside ^b^	Place dies facing down onto a Si wafer and deposit 100 nm PECVD Si_3_N_4_ to insulate backside ^b^	Omitted

^a^ For the 15 µm frontside SOI etch, because the etch depth is sufficiently shallow we now use 1 µm of SPR-700 resist for improved alignment accuracy. ^b^ This step needs to be carefully tested, to avoid any deposition on the front side where the recording sites or metal pads could be impaired by a film of dielectric. Only a thin film should be used, sufficient to provide insulation but not thick enough to risk accidental covering of the front-side. If necessary, a dilute HF dip can be performed to remove any accidental, thin, front-side deposition. ^c^ Optional deposition, used to color-code different wafers.

**Table 3:** Comparison of Electroplating Approaches.

Detail	Packaged Plating	Short-Circuited Breakout Beams	Seed and Mask	Electroless
Method	Electrolytic	Electrolytic	Electrolytic	Electroless
Common metal choices	Au, Ni, Cu	Au, Ni, Cu	Au, Ni, Cu	Ni, Cu
Holder plate design type (see Figure 7)	“A”	“C”	“B”	“A”
Can be plated before packaging	No	Yes	Yes	Yes
Requires further processing after plating	No	Yes	Yes	No
Minimum pitch ^a^	W_pad_ + 2 W_gap_	W_pad_ + 2 W_gap_	W_pad_ + 2 W_gap_	W_pad_ + W_gap_
Requires direct wiring access to plating pads	Yes	Yes	No	No
Advantages	Pads can be plated individually Ability to electrically detect plating endpoint, especially if pads are individually plated	No seed layer or plating mask needed	Controlled plating of all pads in parallel Most common plating method in microfabrication	Tightest pad pitch Aluminum pad compatible Zincate and brief Ni plating can be done on full wafer before assembly
Disadvantages	Devices must be fully packaged before plating Package must be compatible with plating chemicals	Temporary short circuit wiring requires extra space Breaking the short-circuit beams can be difficult Careful resistance balancing needed	Requires chemical etching of mask and seed Mask and seed must be DRIE and O2 plasma ashing compatible	Pad cleanliness is very important, to avoid uneven plating

^a^ W_pad_ is the width of the contact pad and W_gap_ is the distance between the 2-D insert and the contact pad on the holder plate. The minimum pitch is the width of the pad and the spacing. For electrolytic plating, a distance of W_gap_ needs to be covered, so that the minimum spacing to avoid short-circuiting pads is 2 W_gap_. For electroless plating, the gap is bridged from both sides and thus only W_gap_/2 is plated per side, with a minimum spacing of W_gap_.

**Table 4:** Design summary of example designs in this paper.

ID		Design (Part Names)	Recording Site Configuration ^a^	3-D Array Configuration ^b^	Connections per 2-D Insert^c^	Total Connections	Connection Pitch	Device Purpose
1a		B160–F160	2 × 20 @ 13.0 µm	9 × 4	160	1440	60 µm	Conservative design
1b		B160–F20	2 × 2 @ 9.5/14 µm	9 × 40	160	1440	60 µm	Large shank count, tetrode tips
2		B160–F30	1 × 9 @ 250.0 µm	9 × 17	160	1440	60 µm	Optical-only lithography
3	3a	B408–F408	2 × 34 @ 13.0 µm	11 × 6	408	4488	40 µm	Standard design
	3b	B409–F408	2 × 34 @ 13.0 µm	11 × 6	408	4488	40 µm	Compact holder plate
4		B1000–F1006	4 × 42 @ 13.0 µm	10 × 6	1008	10,080	26 µm	Aggressive design
5		B1000–F1010	2 × 50 @ 13.0 µm	10 × 10	1008	10,080	26 µm	Aggressive design
6		B10–F10	1 × 1	8 × 80	149	1192	16.5 to 113.7 µm	Pitch and DRIE etch testing

^a^ Rows/shank × Columns/shank @ Site pitch in µm (identical for columns and rows except 1b). ^b^ Inserts/probe × Shanks/insert. ^c^ For more connections than recording sites per insert (e.g., design 5 and 6), the layout code leaves some connections open, yet the connections are still formed in the electrical assembly. Configurations of the different probes fabricated in this paper. Part names begin with B for the holder plates and F for the 2-D probe inserts. Electrical characteristics are shown in Table 5.

**Table 5:** Electrical Characterization of Probe Components. Measurement accuracy is ±5%, driven by the quality of the contact to the metal pad during measurement, rather than the measurement equipment itself. One measurement was taken per point.

Design	Wire Length (mm)	Wire Aspect Ratio (n_sq_ = L/W)	Resistance (kΩ)	Capacitance (pF)
Holder B10	5.6	1900	1.02	0.56
Insert F10 ^a^	1.9	1050	n/a	0.08
Holder B160	11.3	7200	8.53	1.39
Insert F30	2.1	950	0.29	0.09
Insert F160	4.0	7700	9.30	0.28
Holder B408	13.0	14,200	21.7	1.82
Holder B409	13.9	34,750	55.1	1.28
Insert F408	5.3	8700	9.45	0.48
Holder B1000	15.8	39,400	66.1	1.88
Insert F1006	8.2	14,100	18.1	0.71
Insert F1010	5.4	11,300	12.8	0.63

^a^ No resistance measurement for F10 because only 1 site/shank. Design names correspond to the details shown in Table 4.
